# A novel *TTN* deletion in a family with skeletal myopathy, facial weakness, and dilated cardiomyopathy

**DOI:** 10.1002/mgg3.924

**Published:** 2019-09-05

**Authors:** Jennifer Roggenbuck, Kelly Rich, Ana Morales, Christopher A. Tan, Douglas Eck, Wendy King, Matteo Vatta, Thomas Winder, Bakri Elsheikh, Ray E. Hershberger, John T. Kissel

**Affiliations:** ^1^ The Ohio State University Wexner Medical Center Columbus Ohio; ^2^ Invitae Corporation San Francisco California

**Keywords:** dilated cardiomyopathy, myopathy, *TTN*, variant interpretation

## Abstract

**Background:**

Pathogenic variants in *TTN (OMIM 188840),* encoding the largest human protein, are known to cause dilated cardiomyopathy and several forms of skeletal myopathy. The clinical interpretation of *TTN* variants is challenging, however, due to the frequency of missense changes, variable testing and reporting practices in commercial laboratories, and incomplete understanding of the spectrum of *TTN‐*related disease.

**Methods:**

We report a heterozygous *TTN* deletion segregating in a family with an unusual skeletal myopathy phenotype associated with facial weakness, gait abnormality, and dilated cardiomyopathy.

**Results:**

A novel 16.430 kb heterozygous deletion spanning part of the A‐ and M‐bands of *TTN* was identified in the proband and his symptomatic son, as well as in an additional son whose symptoms were identified on clinical evaluation. The deletion was found to be de novo in the proband.

**Conclusion:**

Pathogenic variants in *TTN* may be an unrecognized cause of skeletal myopathy phenotypes, particularly when accompanied by dilated cardiomyopathy.

## 1 CLINICAL REPORT

A father and son affected with proximal‐predominant muscle weakness were evaluated.

**Figure 1:**
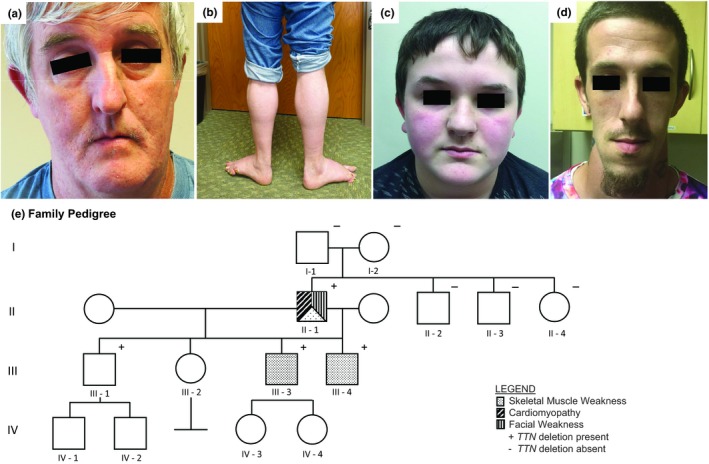
(a) Facial weakness in proband (II‐1); (b) Stance with bilateral foot eversion in proband (II‐1); (c) Facial weakness in son III‐4; (d) Facial weakness in son III‐3; (e) Family pedigree demonstrating vertical transmission of the de novo* TTN* deletion in this family. Paternity was confirmed in the parents of the proband. Son III‐1 was unavailable for clinical evaluation

The proband (II‐1, Figure 1e) initially presented at age 50 with weakness affecting proximal limb, neck flexor, and facial muscles. He reported onset of weakness in childhood, with difficulty running and lifting, and eventually developed a unique gait marked by profound external rotation of bilateral hips. He had mild facial weakness affecting the frontalis and bilateral orbicularis oculi, orbicularis oris, and buccinator muscles (Figure 1a). Electromyography showed a myopathic process; serum creatine kinase was normal. Right bicep muscle biopsy revealed myopathic changes, muscle fiber size variability, and increased internal nuclei. Echocardiogram showed normal left ventricular (LV) size with low normal LV function, with an estimated ejection fraction (EF) of 50%. Genetic testing for fascioscapulohumeral muscular dystrophy, myotonic dystrophy type 2, as well as alpha‐glucosidase testing was normal. A 35‐gene limb‐girdle muscular dystrophy (LGMD) panel from one commercial laboratory identified one missense variant in *TTN*, reported as c.49787T>C, p.Leu16596Pro, and classified as a variant of uncertain significance.

During the course of the proband's evaluation, his 13‐year‐old son (III‐4) was also identified to have muscle weakness. His early motor milestones included crawling at 12 months and walking at 18 months. He reported progressive difficulty with running and lifting. Manual muscle testing identified both proximal (predominant) and distal weakness with asymmetry (L>R), as well as mild facial weakness. He has a high‐arched palate, and ambulates with a bilateral uncompensated gluteus medius gait and is unable to walk on his heels or toes. Echocardiogram at age 15 was normal.

Six years after the proband's initial presentation, a 204‐gene combined neuromuscular and cardiomyopathy panel from a second commercial laboratory identified a partial deletion of *TTN* (exons 346‐362), interpreted as likely pathogenic. All first‐degree relatives were subsequently tested for this variant. Sons III‐1, III‐3, and III‐4 also shared this deletion. No other family members (including both parents of the proband) were found to have the deletion. Clinical evaluation of son III‐3 revealed facial weakness, neck flexor weakness, decreased muscle bulk, as well as proximal and distal weakness with asymmetry (L>R) (Figure 1d). He reported difficulty walking on uneven surfaces. Echocardiogram showed EF of 53%, consistent with borderline‐low systolic function with a LV end‐diastolic dimension at the upper limits of normal. Individual III‐1 declined clinical evaluation. The proband developed heart failure at age 57 (EF 20% with mild‐moderate four‐chamber dilation, meeting criteria for dilated cardiomyopathy [DCM]); he passed away from heart failure at age 58. Clinical characteristics of examined family members are presented in Table 1.

**Table 1:** Clinical characteristics of examined family members with *TTN* deletion

Subject	Gender, age	Onset of weakness	Pattern of weakness	Gait	Asymmetry	Facial weakness	CK	Cardiac phenotype	LVIDd (cm)	LVIDd %ile	EF (%)
II ‐ 1	M, 58	Childhood	Proximal > distal Bilateral hip extensor weakness Bilateral hip flexor weakness Bilateral hip internal rotator weakness Bilateral distal lower extremity weakness	Profound external rotation of bilateral hips “Charlie Chaplin” gait	L>R weakness	+++	Normal	Dilated cardiomyopathy, onset 42y	5.4 cm	<95th %ile	20
III ‐ 3	M, 29	Adulthood	Proximal ≈ distal Bilateral hip flexor weakness Hip internal rotator weakness Bilateral dorsiflexor weakness	Normal	L>R weakness	++	N/A	Borderline‐low ejection fraction	5.6 cm	<95th %tile	53
III ‐ 4	M, 15	Childhood	Proximal > distal Bilateral hip flexor weakness Bilateral dorsiflexor weakness Hip internal rotator weakness Hip external rotator weakness Shoulder girdle weakness	Bilateral uncompensated gluteus medius gait Unable to walk on heels or toes	L>R weakness	+	Normal	Normal	4.8 cm		61

Abbreviations: CK, creatine kinase; LVIDd, left ventricular internal dimension‐diastole; EF, ejection fraction.

## 2 METHODS

Peripheral blood or saliva were evaluated by Next Generation Sequencing (NGS; Lincoln et al., 2015). Each gene was targeted with oligonucleotide baits (Agilent Technologies; Roche; IDT) to capture all coding exons, plus 10–20 bases of flanking intronic sequences, and noncoding regions of clinical interest. Baits were balanced to obtain a minimum of 50× and an average of 350× depth‐of‐sequence read coverage. Titin metatranscript NM_001267550.2 was used as reference sequence. A bioinformatics pipeline incorporated both standard and custom algorithms to identify single‐nucleotide variants, small indels, large indels, structural variants with breakpoints in target sequences, and exon‐level copy number variants (CNVs). In addition to standard GATK‐based analysis (GATK version 3.6**)**, validated coverage‐based CNV detection algorithms designed to flag possible split‐read signals were applied. Once verified, the variant call format was updated and interpreted.

### ETHICAL COMPLIANCE

This work was approved by the Office of Responsible Research Practices at The Ohio State University Wexner Medical Center.

## 3 RESULTS

**Figure 2:**
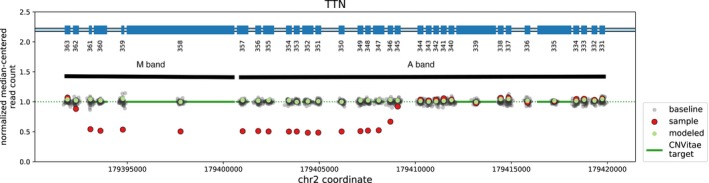
Familial *TTN* deletion NGS reads were used to quantitate copy number of the *TTN* gene. A multiple‐exon deletion in *TTN* was detected. Loss of read count over exons 346‐362 represented by red dots indicative of the comparative loss of properly mapping reads

NGS detected a novel deletion affecting the genomic sequence encompassing coordinates chr2:179392365‐179408795. This deletion is predicted to cause an out‐of‐frame change, including exons 346‐362 as numbered according to metatranscript NM_001267550.2:c.96076_107488del of *TTN*. The variant deleted the last 12 exons of the A‐band and five of six exons in the M‐band (Figure 2). The data obtained by NGS were also confirmed by array CGH. The *TTN* deletion was shown to segregate with a skeletal myopathy phenotype in two additional family members (Figure 1e). Family relationships were confirmed using inference analysis of available single‐nucleotide polymorphisms. This variant, involving exons 346‐362 of the metatranscript, is predicted to affect the long (N2BA) and short (N2B) cardiac isoforms, as well as the long (N2A) skeletal isoform. No additional pathogenic or likely pathogenic *TTN* variants were identified in the tested individuals.

## 4 DISCUSSION

Several *TTN*‐related skeletal myopathies are currently known. Most demonstrate recessive inheritance and do not cause cardiomyopathy, including LGMD2J (a severe, early‐onset LGMD caused by a homozygous 11‐bp deletion/insertion in the M‐line), centronuclear myopathy, and *TTN*‐associated distal myopathy (both caused by recessive truncating or in‐frame deletions/duplications; Savarese, Sarparanta, Vihola, Udd, & Hackman, 2016). Recessive truncating *TTN* variants have also been reported to cause several severe childhood‐onset phenotypes presenting with both skeletal and cardiac muscle disease (Chauveau et al., 2014). Dominant *TTN*‐related skeletal myopathies include tibial muscular dystrophy (caused by the 11‐bp M‐line deletion/insertion in the heterozygous state) and hereditary myopathy with early respiratory failure (caused by dominant missense mutations in exon 344); neither causes cardiac disease (Hackman et al., 2002; Pfeffer & Chinnery, 1993).

Here we report an unusual skeletal myopathy phenotype with facial weakness, gait abnormality and DCM, associated with a novel de novo* TTN* deletion of multiple exons predicted to affect the A‐ and M‐bands of the protein. In this family, the deletion appears to segregate in an autosomal dominant fashion and the natural history of the condition involves childhood‐onset of mild muscle weakness, with additional features of facial weakness and DCM developing in later decades. Although “atypical titinopathies” have been attributed to the presence of second *TTN* variants which modify the expected phenotype (Evila et al., 2014), no significant second *TTN* variants were found in affected family members. The *TTN* missense change initially identified in the proband (reported as c.49787T>C, p.Leu16596Pro, numbered as c.54710T>C, p.Leu18237Pro in NM_001267550.2) has a frequency of 0.11% in Europeans (Lek et al., 2016), was considered likely benign by the second laboratory, and was not present in his affected sons. Other authors have suggested that *TTN* variants may produce both skeletal and cardiac muscle disease in a dominant fashion (Ceyhan‐Birsoy et al., 2013).

The proband's diagnostic odyssey illustrates the challenges associated with NGS technology and variant interpretation. Two different NGS panels were applied; both included *TTN* sequencing and yielded inconsistent results. It is important that clinicians understand the variability between laboratories in detecting and reporting deletions, that the same variant may be interpreted or reported differently between laboratories, and reporting practices within the same laboratory may change over time. This issue is particularly acute in *TTN* and may lead to under‐recognition of clinically significant *TTN* variants and missed diagnoses.

Further data are needed regarding the frequency and pathogenicity of structural variants in *TTN*, which may impact the approach to genetic evaluation for both DCM and skeletal myopathy. We suggest that *TTN* be considered in the genetic differential diagnosis in any patient with both skeletal muscle weakness and DCM.

## CONFLICT OF INTEREST

C. Tan, M. Vatta and T. Winder are employees of Invitae Corporation. B. Elsheikh is a medical consultant f or Biogen Inc. J. Roggenbuck, K. Rich, A. Morales, D. Eck, W. King, R. Hershberger, and J. Kissel report no disclosures.

## PATIENT CONSENT

A signed Patient Consent‐to‐Disclose Form has been obtained for photos/videos of any recognizable patient.
