# Supplementary material for: A novel TTN deletion in a family with skeletal myopathy, facial weakness, and dilated cardiomyopathy
Source: Mol Genet Genomic Med. 2019 Sep 5;7(11):e924. doi: 10.1002/mgg3.924 (PMC6825852; doi:10.1002/mgg3.924)
Supplement: Supplementary file 1 [file MGG3-7-e924-s001.docx]

*TTN* Variants Identified in Affected, Examined Family Members

| **Individual II-1** | | | | |
| --- | --- | --- | --- | --- |
| **Variant** | **Protein Effect** | **Zygosity** | **Population Frequency** | **Classification** |
| NM_001267550.2:c.96076_107488del | Exons 346-362 | heterozygous | Not observed | Likely Pathogenic |
| NM_001267550.2:c.54710T>C | p.Leu18237Pro | heterozygous | 0.05% | Likely Benign |
| NM_001267550.2:c.100579G>A | p.Val33527Ile | homozygous | 3.10% | Benign |
| NM_001267550.2:c.10256G>A | p.Ser3419Asn | homozygous | 85.10% | Benign |
| NM_001267550.2:c.102833G>T | p.Gly34278Val | homozygous | 3.90% | Benign |
| NM_001267550.2:c.105384A>G | Silent | homozygous | 35.20% | Benign |
| NM_001267550.2:c.105582C>T | Silent | homozygous | 4.40% | Benign |
| NM_001267550.2:c.105782C>T | p.Pro35261Leu | homozygous | 4.00% | Benign |
| NM_001267550.2:c.107267T>C | p.Val35756Ala | homozygous | 4.50% | Benign |
| NM_001267550.2:c.11252G>A | p.Gly3751Asp | homozygous | 85.10% | Benign |
| NM_001267550.2:c.12780G>A | Silent | heterozygous | 16.50% | Benign |
| NM_001267550.2:c.14610C>T | Silent | homozygous | 99.50% | Benign |
| NM_001267550.2:c.15792T>C | Silent | heterozygous | 5.40% | Benign |
| NM_001267550.2:c.22384G>C | p.Asp7462His | heterozygous | 17.90% | Benign |
| NM_001267550.2:c.2244G>A | Silent | homozygous | 31.90% | Benign |
| NM_001267550.2:c.23099-3T>C | Intronic | heterozygous | 32.30% | Benign |
| NM_001267550.2:c.23223G>A | Silent | homozygous | 99.50% | Benign |
| NM_001267550.2:c.24431A>C | p.Glu8144Ala | heterozygous | 17.70% | Benign |
| NM_001267550.2:c.25064C>A | p.Ala8355Glu | heterozygous | 30.90% | Benign |
| NM_001267550.2:c.25274G>A | p.Ser8425Asn | heterozygous | 17.80% | Benign |
| NM_001267550.2:c.26091A>T | Silent | heterozygous | 30.60% | Benign |
| NM_001267550.2:c.26289A>G | Silent | heterozygous | 32.00% | Benign |
| NM_001267550.2:c.26408A>G | p.Asn8803Ser | heterozygous | 17.90% | Benign |
| NM_001267550.2:c.26655C>T | Silent | heterozygous | 30.60% | Benign |
| NM_001267550.2:c.31564A>G | p.Ile10522Val | heterozygous | 35.40% | Benign |
| NM_001267550.2:c.31864G>A | p.Gly10622Arg | heterozygous | 39.90% | Benign |
| NM_001267550.2:c.33287G>A | p.Arg11096His | heterozygous | 23.50% | Benign |
| NM_001267550.2:c.33834G>A | Silent | heterozygous | 26.00% | Benign |
| NM_001267550.2:c.3601A>G | p.Lys1201Glu | homozygous | 69.40% | Benign |
| NM_001267550.2:c.3884C>T | p.Ser1295Leu | homozygous | 96.10% | Benign |
| NM_001267550.2:c.4480+6C>T | Intronic | homozygous | 95.90% | Benign |
| NM_001267550.2:c.56101A>G | p.Asn18701Asp | heterozygous | 35.50% | Benign |
| NM_001267550.2:c.57315T>C | Silent | heterozygous | 23.50% | Benign |
| NM_001267550.2:c.61245A>G | Silent | heterozygous | 35.40% | Benign |
| NM_001267550.2:c.62058T>C | Silent | heterozygous | 35.40% | Benign |
| NM_001267550.2:c.64208C>T | p.Thr21403Ile | heterozygous | 35.10% | Benign |
| NM_001267550.2:c.65682A>G | Silent | heterozygous | 35.90% | Benign |
| NM_001267550.2:c.67075G>A | p.Val22359Ile | heterozygous | 24.20% | Benign |
| NM_001267550.2:c.67246G>C | p.Ala22416Pro | homozygous | 99.90% | Benign |
| NM_001267550.2:c.70830C>T | Silent | heterozygous | 24.00% | Benign |
| NM_001267550.2:c.76343G>A | p.Ser25448Asn | heterozygous | 4.10% | Benign |
| NM_001267550.2:c.78674T>C | p.Ile26225Thr | heterozygous | 23.70% | Benign |
| NM_001267550.2:c.79062T>A | Silent | heterozygous | 4.60% | Benign |
| NM_001267550.2:c.79862C>T | p.Thr26621Met | heterozygous | 23.40% | Benign |
| NM_001267550.2:c.83323A>G | p.Ile27775Val | heterozygous | 35.40% | Benign |
| NM_001267550.2:c.83673T>C | Silent | heterozygous | 35.10% | Benign |
| NM_001267550.2:c.88187T>C | p.Ile29396Thr | heterozygous | 35.10% | Benign |
| NM_001267550.2:c.97795+6G>T | Intronic | homozygous | 38.30% | Benign |
| NM_001267550.2:c.9781G>A | p.Val3261Met | homozygous | 85.00% | Benign |
| NM_001267550.2:c.98164A>T | p.Ile32722Phe | homozygous | 2.50% | Benign |
| NM_001267550.2:c.9879A>G | Silent | homozygous | 99.20% | Benign |

| **Individual III-1** | | | | |
| --- | --- | --- | --- | --- |
| **Variant** | **Protein Effect** | **Zygosity** | **Population Frequency** | **Classification** |
| NM_001267550.2:c.96076_107488del | Exons 346-362 | heterozygous | Not observed | Likely Pathogenic |
| NM_001267550.2:c.81617T>C | p.Ile27206Thr | heterozygous | 0.00% | Uncertain Significance |
| NM_001267550.2:c.10256G>A | p.Ser3419Asn | homozygous | 85.10% | Benign |
| NM_001267550.2:c.11252G>A | p.Gly3751Asp | homozygous | 85.10% | Benign |
| NM_001267550.2:c.14610C>T | Silent | homozygous | 99.50% | Benign |
| NM_001267550.2:c.2244G>A | Silent | heterozygous | 31.90% | Benign |
| NM_001267550.2:c.23223G>A | Silent | homozygous | 99.50% | Benign |
| NM_001267550.2:c.2432C>T | p.Thr811Ile | heterozygous | 16.90% | Benign |
| NM_001267550.2:c.31864G>A | p.Gly10622Arg | heterozygous | 39.90% | Benign |
| NM_001267550.2:c.33287G>A | p.Arg11096His | heterozygous | 23.50% | Benign |
| NM_001267550.2:c.3601A>G | p.Lys1201Glu | homozygous | 69.40% | Benign |
| NM_001267550.2:c.3884C>T | p.Ser1295Leu | homozygous | 96.10% | Benign |
| NM_001267550.2:c.4480+6C>T | Intronic | homozygous | 95.90% | Benign |
| NM_001267550.2:c.57315T>C | Silent | heterozygous | 23.50% | Benign |
| NM_001267550.2:c.67246G>C | p.Ala22416Pro | homozygous | 99.90% | Benign |
| NM_001267550.2:c.70830C>T | Silent | heterozygous | 24.00% | Benign |
| NM_001267550.2:c.78674T>C | p.Ile26225Thr | heterozygous | 23.70% | Benign |
| NM_001267550.2:c.92901C>T | Silent | heterozygous | 1.90% | Benign |
| NM_001267550.2:c.9781G>A | p.Val3261Met | homozygous | 85.00% | Benign |
| NM_001267550.2:c.9879A>G | Silent | homozygous | 99.20% | Benign |

| **Individual III-3** | | | | |
| --- | --- | --- | --- | --- |
| **Variant** | **Protein Effect** | **Zygosity** | **Population Frequency** | **Classification** |
| NM_001267550.2:c.96076_107488del | Exons 346-362 | heterozygous | Not observed | Likely Pathogenic |
| NM_001267550.2:c.55374C>G | p.Ser18458Arg | heterozygous | 0.03% | Uncertain Significance |
| NM_001267550.2:c.10256G>A | p.Ser3419Asn | homozygous | 85.10% | Benign |
| NM_001267550.2:c.11252G>A | p.Gly3751Asp | homozygous | 85.10% | Benign |
| NM_001267550.2:c.14610C>T | Silent | homozygous | 99.50% | Benign |
| NM_001267550.2:c.2244G>A | Silent | heterozygous | 31.90% | Benign |
| NM_001267550.2:c.23223G>A | Silent | homozygous | 99.50% | Benign |
| NM_001267550.2:c.24516C>T | Silent | heterozygous | 9.00% | Benign |
| NM_001267550.2:c.31864G>A | p.Gly10622Arg | heterozygous | 39.90% | Benign |
| NM_001267550.2:c.33287G>A | p.Arg11096His | heterozygous | 23.50% | Benign |
| NM_001267550.2:c.3601A>G | p.Lys1201Glu | homozygous | 69.40% | Benign |
| NM_001267550.2:c.3884C>T | p.Ser1295Leu | homozygous | 96.10% | Benign |
| NM_001267550.2:c.42958A>G | p.Lys14320Glu | heterozygous | 8.30% | Benign |
| NM_001267550.2:c.4480+6C>T | Intronic | homozygous | 95.90% | Benign |
| NM_001267550.2:c.57315T>C | Silent | heterozygous | 23.50% | Benign |
| NM_001267550.2:c.67246G>C | p.Ala22416Pro | homozygous | 99.90% | Benign |
| NM_001267550.2:c.70830C>T | Silent | heterozygous | 24.00% | Benign |
| NM_001267550.2:c.7545C>T | Silent | heterozygous | 9.20% | Benign |
| NM_001267550.2:c.78674T>C | p.Ile26225Thr | heterozygous | 23.70% | Benign |
| NM_001267550.2:c.9781G>A | p.Val3261Met | homozygous | 85.00% | Benign |
| NM_001267550.2:c.9879A>G | Silent | homozygous | 99.20% | Benign |

| **Individual III-4** | | | | |
| --- | --- | --- | --- | --- |
| **Variant** | **Protein Effect** | **Zygosity** | **Population Frequency** | **Classification** |
| NM_001267550.2:c.96076_107488del | Exons 346-362 | heterozygous | Not observed | Likely Pathogenic |
| NM_001267550.2:c.102519C>T | Silent | homozygous | 18.20% | Benign |
| NM_001267550.2:c.10256G>A | p.Ser3419Asn | homozygous | 85.10% | Benign |
| NM_001267550.2:c.103781G>A | p.Arg34594His | homozygous | 17.50% | Benign |
| NM_001267550.2:c.104988C>T | Silent | homozygous | 18.20% | Benign |
| NM_001267550.2:c.105180G>C | p.Glu35060Asp | homozygous | 3.80% | Benign |
| NM_001267550.2:c.105384A>G | Silent | homozygous | 35.20% | Benign |
| NM_001267550.2:c.11252G>A | p.Gly3751Asp | homozygous | 85.10% | Benign |
| NM_001267550.2:c.12780G>A | Silent | heterozygous | 16.50% | Benign |
| NM_001267550.2:c.14610C>T | Silent | homozygous | 99.50% | Benign |
| NM_001267550.2:c.22384G>C | p.Asp7462His | heterozygous | 17.90% | Benign |
| NM_001267550.2:c.2244G>A | Silent | heterozygous | 31.90% | Benign |
| NM_001267550.2:c.23099-3T>C | Intronic | heterozygous | 32.30% | Benign |
| NM_001267550.2:c.23223G>A | Silent | homozygous | 99.50% | Benign |
| NM_001267550.2:c.24431A>C | p.Glu8144Ala | heterozygous | 17.70% | Benign |
| NM_001267550.2:c.25064C>A | p.Ala8355Glu | heterozygous | 30.90% | Benign |
| NM_001267550.2:c.25274G>A | p.Ser8425Asn | heterozygous | 17.80% | Benign |
| NM_001267550.2:c.26091A>T | Silent | heterozygous | 30.60% | Benign |
| NM_001267550.2:c.26289A>G | Silent | heterozygous | 32.00% | Benign |
| NM_001267550.2:c.26408A>G | p.Asn8803Ser | heterozygous | 17.90% | Benign |
| NM_001267550.2:c.26655C>T | Silent | heterozygous | 30.60% | Benign |
| NM_001267550.2:c.28313G>A | p.Arg9438Gln | heterozygous | 4.50% | Benign |
| NM_001267550.2:c.31564A>G | p.Ile10522Val | heterozygous | 35.40% | Benign |
| NM_001267550.2:c.31864G>A | p.Gly10622Arg | heterozygous | 39.90% | Benign |
| NM_001267550.2:c.33287G>A | p.Arg11096His | heterozygous | 23.50% | Benign |
| NM_001267550.2:c.33834G>A | Silent | heterozygous | 26.00% | Benign |
| NM_001267550.2:c.3601A>G | p.Lys1201Glu | heterozygous | 69.40% | Benign |
| NM_001267550.2:c.3884C>T | p.Ser1295Leu | homozygous | 96.10% | Benign |
| NM_001267550.2:c.4480+6C>T | Intronic | homozygous | 95.90% | Benign |
| NM_001267550.2:c.56101A>G | p.Asn18701Asp | heterozygous | 35.50% | Benign |
| NM_001267550.2:c.57315T>C | Silent | heterozygous | 23.50% | Benign |
| NM_001267550.2:c.58436G>A | p.Arg19479His | heterozygous | 17.60% | Benign |
| NM_001267550.2:c.59585C>T | p.Pro19862Leu | heterozygous | 17.70% | Benign |
| NM_001267550.2:c.61245A>G | Silent | heterozygous | 35.40% | Benign |
| NM_001267550.2:c.62058T>C | Silent | heterozygous | 35.40% | Benign |
| NM_001267550.2:c.64208C>T | p.Thr21403Ile | heterozygous | 35.10% | Benign |
| NM_001267550.2:c.65682A>G | Silent | heterozygous | 35.90% | Benign |
| NM_001267550.2:c.67075G>A | p.Val22359Ile | heterozygous | 24.20% | Benign |
| NM_001267550.2:c.67246G>C | p.Ala22416Pro | homozygous | 99.90% | Benign |
| NM_001267550.2:c.70830C>T | Silent | heterozygous | 24.00% | Benign |
| NM_001267550.2:c.74839C>T | p.Arg24947Cys | heterozygous | 17.50% | Benign |
| NM_001267550.2:c.78674T>C | p.Ile26225Thr | heterozygous | 23.70% | Benign |
| NM_001267550.2:c.79862C>T | p.Thr26621Met | heterozygous | 23.40% | Benign |
| NM_001267550.2:c.83323A>G | p.Ile27775Val | heterozygous | 35.40% | Benign |
| NM_001267550.2:c.83673T>C | Silent | heterozygous | 35.10% | Benign |
| NM_001267550.2:c.88187T>C | p.Ile29396Thr | heterozygous | 35.10% | Benign |
| NM_001267550.2:c.93243C>T | Silent | heterozygous | 18.40% | Benign |
| NM_001267550.2:c.97613G>A | p.Arg32538His | homozygous | 17.80% | Benign |
| NM_001267550.2:c.97795+6G>T | Intronic | homozygous | 38.30% | Benign |
| NM_001267550.2:c.9781G>A | p.Val3261Met | homozygous | 85.00% | Benign |
| NM_001267550.2:c.98098+9T>A | Intronic | homozygous | 18.30% | Benign |
| NM_001267550.2:c.982C>T | p.Arg328Cys | heterozygous | 12.40% | Benign |
| NM_001267550.2:c.9879A>G | Silent | homozygous | 99.20% | Benign |
